# Quality of life in the workplace for nursing staff at public healthcare institutions**^1^**

**DOI:** 10.1590/1518-8345.1149.2713

**Published:** 2016-08-08

**Authors:** María Olga Quintana Zavala, Tatiana Paravic Klinj, Katia Lorena Saenz Carrillo

**Affiliations:** 2 PhD, Full Professor, Departamento de Enfermería, Universidad de Sonora, Hermosillo, Sonora, Mexico.; 3 PhD, Full Professor, Facultad de Enfermería, Universidad de Concepción, Concepción, VIII Región, Chile.; 4 PhD, Associate Profesor, Facultad de Ciencias Físicas y Matemáticas, Universidad de Concepción, Concepción, VIII Región, Chile

**Keywords:** Quality of Life, Work, Nursing Staff

## Abstract

**Objective:**

to determine the quality of life in the workplace for nursing staff at public institutions in Hermosillo, Sonora, Mexico.

**Method:**

quantitative, correlational, cross-sectional, and comparative. We used a probabilistic sample of 345 nurses with data collected in 2013 using an instrument created by the authors to gather bio-socio-demographic data and the CVT-GOHISALO instrument with a Cronbach's alpha of 0.95. SPSS 15 was used to analyze the data. A Kolmogorov-Smirnov test was used to calculate the normality of the data; the medians were compared using the Mann-Whitney U test and Kruskal-Wallis test with the significance level set at 0.05.

**Results:**

the average overall quality of life in the workplace for nursing staff was 207.31 (DE 41.74), indicating a moderate level. The quality of life in the workplace was higher for people with permanent contracts (p=0.007) who did not engage in other remunerative activities (p=0.046). Differences in the quality of life in the workplace were observed depending on the institution where the subjects worked (p=0.001).

**Conclusion:**

the nursing staff perceives itself as having a moderate-level quality of life in the workplace. This level was determined in the statistical analysis based on the type of contract, whether the person performed other remunerated activities, and the institution where the person worked.

## Introduction

Quality of life in the workplace is a multidimensional concept that applies when the employee is able to cover the following personal necessities through employment or his or her own ventures: institutional support, security and integration into his or her role at work and satisfaction related to this role, a sense of wellbeing obtained through his or her work and the personal development achieved, and the management of his or her free time^1^. This concept has been used with increasing frequency to describe certain environmental and humanistic values that are neglected by industrial societies in their pursuit of technological advancement, productivity, and economic growth^2^.

Among healthcare professionals, quality of life in the workplace ought to meet the highest standards because these professionals appear to have the knowledge and the means necessary to avoid risks and perform self-care activities in all areas. However, various studies underscore that this ideal is far from the reality. Convincing evidence exists that healthcare professionals are faced with various problems.

In the specific case of nursing, employees have developed pathologies including Burnout Syndrome, workplace stress, conflicts related to violence within healthcare institutions (directed at both clients and the nursing staff itself), indices of poor workplace satisfaction, and depression. This situation could be associated with workplace conditions related to the types of contracts, employees having two or more jobs, and the type of institution where a given employee works, among other factors. The issues could also be related to risks recognized in the literature related to hospital work, which involves a mental workload that directly impacts the quality of care, quality of life in the workplace, and overall quality of life^3^ ^-^ ^8^. 

The quality of life of nursing employees in the workplace has been studied worldwide. Diverse work-related issues are notable across all of Latin America, including unstable employment, inadequate workplace conditions, limited availability of equipment and materials that are essential to effectively improve the quality of care, overwork related to the scarcity of nursing staff, devaluation of the nursing profession, and expectations that nursing professionals will migrate^9^ ^-^ ^16^.

In northwestern Mexico, the social evidence of this problem can be seen in protests related to current workplace conditions for nursing staff. Therefore, it is especially important to understand the level of the quality of life in the workplace of those who are professionally responsible for the health of the population.

This study has the general objective of determining the quality of life in the workplace of nursing staff employed in public healthcare institutions in Hermosillo, Sonora, Mexico. The following hypotheses are proposed:

- Nursing staff with permanent contracts have a better overall quality of life in the workplace than nursing staff who have fixed-term contracts.

- The quality of life of nursing employees who perform another remunerated activity is lower than that of employees who do not perform another remunerated activity. 

- Overall quality of life in the workplace is perceived differently depending on the institution in which a nursing employee works.

## Methods

Design type: Quantitative, correlational, cross-sectional, and comparative.

Unit of analysis: Nursing staff who work in seven public healthcare institutions in a city in the northeast of Mexico.

Population: A total of 1503 nursing employees.

Sample: Probabilistic, stratified by level of care (first, second, and third). Based on a 5% error rate and a 10% loss rate, the sample size was 345 members of the nursing staff. The sample was collected in 2013.

Data collection instruments: a) A questionnaire addressing bio-socio-demographic background characteristics and b) the CVT-GOHISALO instrument validated for the Mexican population^17^.

Questionnaire addressing bio-socio-demographic background: Fifteen items developed by the researchers were included to collect bio-socio-demographic and work-related variables from the nursing staff. The variables were sex, age, relationship status, place of origin, existence of children, institution (place of work), role performed (workplace duties), type of service performed, shifts worked, existence of another remunerated activity, type of contract, category of nurse, and medical licenses obtained in the previous six months.

CVT-GOHISALO instrument: This instrument was used to measure the quality of life in the workplace. The instrument was created by researchers at the Occupational Health Research Institute (Instituto de Investigación en Salud Ocupacional) of Guadalajara, Mexico and validated for the Mexican population. It consists of 74 items that represent indicators grouped into sub-dimensions that are themselves composed of 7 dimensions. The average of these dimensions is considered the overall perceived quality of life in the workplace score and is classified as follows: low= 56-191, moderate= 192-227, and high= 228-296. According to the Cronbach's alpha indicator, the instrument's reliability for this study was 0.95. 

Data collection procedure: The data in this study were collected in nursing employees' workplaces (7 public healthcare institutions). The instrument was self-administered, and the response time was approximately 15 minutes.

Statistical analysis plan: SPSS version 15 was used for the statistical analysis. The normality of the data was demonstrated using the Kolmogorov-Smirnov test. Using the results, medians were compared from a non-parametric perspective using the Mann-Whitney U test and Kruskal-Wallis test. The significance level was set at 0.05.

Ethical considerations: This study was conducted with the authorization of the Ethics Committee of the University of Concepción Medical School in Chile and the authorization of the Ethics Committees from the participating healthcare institutions in a city in the northeast of Mexico (Codes 162/UNISON/162/07/12, 051/12, and SSS/CEO/EYC/2012/2013). Additionally, an informed consent letter was provided to each of the participants. Ezequiel Emmanuel's seven ethical requirements were taken into account throughout the development of the study^18^. 

## Results

The sample primarily was female (79.4%), was professional nurses (73%), was with a romantic partner (64.3%), was between 19 and 39 years of age (68.5%), had children (67%), and was of local origin (66.1%). The majority performed duties involving direct client service (87%), worked in hospitalization services (35.4%), worked morning shifts (52.5%), did not perform other remunerated activities (86.7%), had permanent contracts (69.9%), and had not obtained a medical license in the previous six months (67.5%). This information is presented in Table 1.

**Table 1:** Nursing staff according to sociodemographic and work-related variables, Hermosillo, Sonora, Mexico, 2013.

Variables		n (%)
Sex		
	Female	274 (79.4%)
	Male	71 (20.6%)
Age		
	19 to 39 years	237 (68.5)
	40 to 60 years	108 (31.3%)
Relationship status		
	Has a partner	222 (64.3%)
	Does not have a partner	123 (35.7%)
Place of origin		
	Local	228 (66.1%)
	Not local	117 (33.9%)
Has children		
	Yes	231 (67.0%)
	No	114 (33.0%)
Institution		
	A	83 (24.1%)
	B	9 (2.6%)
	C	103 (2.09%)
	D	81 (23.5%)
	E	24 (7.0%)
	F	11(3.2%)
	G	34 (9.9%)
Role performed		
	Direct care	300 (87%)
	Administrative	45 (13%)
Service provided		
	Consultation	57 (16.5%)
	Hospitalization	122 (35.4%)
	Intensive Care Unit/Surgical Urgent Care Unit	93 (27.0%)
	Other	73 (21.2%)
Shift		
	Morning	181 (52.5%)
	Overnight	57 (16.5%)
	Evening	66 (19.1%)
	Weekends and holidays*	41 (11.9%)
Performs another remunerated activity		
	Yes	46 (13.3%)
	No	299 (86.7%)
Type of contract		
	Fixed-term contract	104 (30.1%)
	Permanent	241 (69.9%)
Category of nurse		
	Professional	253 (73%)
	Non-professional	92 (27%)
Medical licenses obtained in the previous six months		
	Yes	112 (32.5%)
	No	233 (67.5%)

N=345

* Refers to the current form of shift recognized in Mexico known as a "Jornada", which includes Saturdays, Sundays, and Holidays.

The average overall quality of life in the workplace for the nursing staff was 207.31 (SD 41.74), which indicated a moderate level. 

The first hypothesis is that nursing staff with permanent contracts have a better overall quality of life in the workplace than nursing staff with fixed-term contracts. We rejected the null hypothesis with the value p=0.007 and observed that overall quality of life in the workplace for the nursing staff according to the type of contract was higher for people with permanent contracts compared with those who had fixed-term contracts (Table 2). 

**Table 2:** Mann-Whitney U test for overall quality of life in the workplace for nursing staff according to the type of contract, Hermosillo, Sonora, Mexico, 2013.

Variable	Type of contract	N*	Mean	S.D.	Median	Mann-Whitney U test	p-value
Overall quality of life in the workplace for nursing staff	Fixed-term	104	198.84	39.44	200.50	10.223	.007†
	Permanent	241	210.97	42.26	219.00		
	Total	345	207.31	41.74	210.00		

*n=345; †p≤0.05

The second hypothesis is that nursing staff who perform another remunerated activity have a lower overall quality of life in the workplace than those who do not perform another remunerated activity. The null hypothesis was rejected with the value p=0.046. We observed that the overall quality of life in the workplace for nursing staff was greater among nursing employees who did not perform another type of remunerated activity compared with nursing employees who did perform another type of remunerated activity. This finding is shown in Table 3.

**Table 3:** Mann-Whitney U test for overall quality of life in the workplace for nursing staff according to the variable regarding the performance of another remunerated activity, Hermosillo, Sonora, Mexico, 2013.

Variable	Performs another remunerated activity	N*	Mean	S.D.	Median	Mann-Whitney U test	p-value
Employees' perceived overall quality of life in the workplace	Yes	46	195.67	40.83	197.50	5.600	.046†
	No	299	209.10	41.66	213.00		
	Total	345	207.31	41.74	210.00		

*n=345; †p≤0.05

The third hypothesis suggests the existence of different levels of overall quality of life in the workplace depending on the institution where the nursing employee works. The null hypothesis was rejected with the value p=0.001 because there was evidence that the overall quality of life in the workplace for nursing staff differed according to the institution where an employee worked. A high overall quality of life in the workplace was identified for those who worked for Institution F, whereas a low overall quality of life in the workplace was identified for those who worked for Institution B. This information is presented in Table 4.

**Table 4:** Kruskal-Wallis test of overall quality of life in the workplace for nursing staff according to the institution where an employee works, Hermosillo, Sonora, Mexico, 2013

Variable	Institution	N*	Mean	S.D.	Median	X**^2^**	gl	p-value
Overall quality of life in the workplace for nursing staff	A	83	218.07	42.73	222.00	21.905	6	.001†
	B	9	192.33	12.47	192.00			
	C	103	196.05	42.27	199.00			
	D	81	210.41	41.08	214.00			
	E	24	207.95	44.20	214.00			
	F	11	237.09	17.42	237.00			
	G	34	201.64	37.73	200.50			

*n=345; †p≤0.001

## Discussion

This study found that the nursing staff were moderately satisfied with their overall quality of life in the workplace. This finding is consistent with the results obtained in other related studies in which the majority of the nursing staff display moderate levels of satisfaction with their quality of life in the workplace^15^ ^,^ ^19^. Taking quality of life in the workplace as a strong indicator of human experiences in the workplace and the degree of satisfaction experienced by the people who perform the work^20^, the strongest indicator of dissatisfaction observed was related to wages and contractual rights. Therefore, it is essential for the nursing staff to be compensated adequately for their contributions to enable their quality of life to be a true reflection of their work's contribution to society.

Similarly, improving the quality of life in the workplace for nursing staff can help institutions retain the nursing workforce. This issue should be considered by human resources administrators. In this vein, we must emphasize that establishing programs to improve quality of life in the workplace for the nursing staff can improve organizational effectiveness because quality of life is a predictor of this effectiveness^14^. Accordingly, attending to the needs of the nursing staff should be a priority for the healthcare system because it represents a quality standard that has direct repercussions for the people receiving care. The healthcare system (i.e., the body charged with caring for the nursing staff) is responsible for making demands of nurses, getting the most out of their work, providing them with a decent salary, and making it possible for them to receive consistent training^19^. 

Therefore, studying the quality of life in the workplace for nursing employees is of utmost importance for the healthcare system, the nursing staff, and those who use their services because the results provide opportunities to implement strategies to elevate the occupational health of the visible nursing staff. Healthy environments should be promoted in the workplace, such as environments that allow the nursing staff to have the tools they need to provide quality healthcare to their patients. This goal will only be accomplished when healthcare institutions provide broad institutional support and workplace security and support integration into the position, satisfaction, professional development, individual wellbeing, and work-life balance. In terms of resilience, this approach will foster greater confidence in the healthcare system on the part of both the nursing staff and the users of health services and promote greater cooperation in working toward shared objectives^21^.

The overall quality of life in the workplace for the nursing staff evaluated according to the type of contract was greater among those who had permanent contracts than among those with fixed-term contracts. This situation affects 30% of the participants in this study, especially the younger participants (63% of the group aged 19-29 years have a fixed-term contract) for whom the process of achieving greater workplace stability is increasingly difficult due to the dearth of new job openings in developing countries in Latin America^22^ ^-^ ^23^.

Low- or mid-level quality of life in the workplace for nursing staff can be associated with the intention of leaving the profession. Therefore, the likelihood of nurses seeking other work and deciding to leave the profession will decline if the quality of life in the workplace is improved for the nursing staff. This goal will require an organizational change aimed at adopting a different form of work using the paradigm of quality of life in the workplace^24^.

In this study, 13% of the members of the nursing team performed another type of remunerated activity. This finding is worrisome because having two formal jobs requires physical and psychological sacrifices that lead to greater economic remuneration and also to reduced free time available for leisure and recreational activities; additionally, this situation leads to fatigue, can have repercussions for the employee's health and may be directly related to errors on the job. This issue creates a dangerous situation for nursing team members, both for themselves and for the clients for whom they provide care; there is also evidence of a greater number of depression and stress cases among nursing employees who work a double shift. This finding is a red flag that must be addressed because a double or triple shift is thought to directly impact female employees' health and, according to our observations in this investigation and related studies, nursing continues to be a profession primarily practiced by women^25^.

The overall quality of life in the workplace also varied significantly according to the healthcare institution where the employee worked. The averages indicated that the nursing staff who worked for Institution F had the highest overall quality of life in the workplace, which was in contrast with those who worked for Institution B, where the overall quality of life in the workplace was generally low. This result can be attributed to the characteristics of the institutions evaluated even though they all belonged to the public sector. Specifically comparing the two institutions with contrasting results (highest and lowest averages for quality of life among all institutions) reveals differences. For instance, institution F has cutting-edge technological equipment, modern facilities, pleasant workplace environment and climate, adequate lighting, multiple entrances, a parking lot, a nursing staff made up entirely of professionals, and rest areas specifically designated for nurses. Nurses work only morning and evening shifts because there are no hospitalization areas and outpatient care is provided in areas for external consultations and treatment, which are scheduled from Monday to Friday.

In contrast, Institution B is a rehabilitation hospital that does not require high-tech equipment but does need to be renovated in terms of furniture (i.e., beds and shelves) and also needs better lighting, ventilation, and larger green areas due to the characteristics of the patients and families receiving care. Another characteristic that could have an effect is the category of nurses because a significant portion of the nursing staff in Institution B is not professionalized. These employees perform nursing activities that are not differentiated from those of the professional staff, which can lead to performance errors and frustration among employees.

Therefore, it is necessary to offer users of public healthcare services facilities and support services of the highest possible quality to achieve better working conditions for nurses, doctors, and other public sector support staff.

## Conclusion

This study demonstrated that both nursing staff who have fixed-term contracts and those who perform other remunerated activities have on average a lower overall quality of life in the workplace than employees who have permanent contracts and who do not perform other remunerated activities. Additionally, the study showed that differences exist in the average overall quality of life in the workplace depending on the healthcare institution where an employee works. These averages range from high to moderate to a low quality of life in the workplace, which can be attributed to differences in the healthcare institutions' characteristics, among other variables.

## Recommendations

Promote lines of research that deepen the understanding of quality of life in the workplace for nursing staff and deploy strategies and programs directed at improvement. In the long term, this approach will translate into improvements in workplace satisfaction and the quality of care provided by the nursing staff.
